# Screening and optimization of indole-3-acetic acid production and phosphate solubilization by rhizobacterial strains isolated from *Acacia cyanophylla* root nodules and their effects on its plant growth

**DOI:** 10.1186/s43141-020-00090-2

**Published:** 2020-11-11

**Authors:** Sara Lebrazi, Karsten Niehaus, Hanna Bednarz, Mouhcine Fadil, Marwa Chraibi, Kawtar Fikri-Benbrahim

**Affiliations:** 1 Laboratory of Microbial Biotechnology, Faculty of Sciences and Technology, Sidi Mohamed Ben Abdellah University, P.O. Box 2202, Fez, Morocco; 2 Proteome and Metabolome Research, Faculty of Biology Center for Biotechnology (CeBiTec), Bielefeld, Germany; 3 Physico-Chemical Laboratory of Inorganic and Organic Materials, Materials Science Center (MSC), Ecole Normale Supérieure, Mohammed V University in Rabat, Rabat, Morocco

**Keywords:** PGPR, Optimization, Indole-3-acetic acid production, Phosphate solubilization, *Acacia cyanophylla*

## Abstract

**Background:**

Plant growth-promoting rhizobacteria (PGPR) are known to improve plant growth and are used as biofertilizers, thanks to their numerous benefits to agriculture such as phosphorus solubilization and phytohormone production. In this paper, four rhizospheric bacteria (*Phyllobacterium* sp., *Bacillus* sp., *Agrobacterium* sp., and *Rhizobium* sp.) isolated from surface-sterilized root nodules of *Acacia cyanophylla* were tested for their ability to solubilize inorganic phosphate and to produce indole-3-acetic acid (IAA) under laboratory conditions. Then, the best IAA producer (*Rhizobium* sp.) was selected to test optimized conditions for IAA production. Finally, the effect of the four strains on plant growth for *A. cyanophylla* was evaluated in vivo.

**Results:**

The results showed that the totality of the tested isolates had solubilized inorganic phosphate (P) in both NBRIP (National Botanical Research Institute Phosphate) and PVK (Pikovskaya) media. *Bacillus* sp. was a high P-solubilizer and showed maximum solubilization in PVK (519 μg ml^-1^) and NBRIP (782 μg ml^-1^). The optimization of maximum phosphate solubilization was done using different sources of carbon (1%) and nitrogen (0.1%). Glucose and ammonium sulfate were selected to be the best carbon and nitrogen source for phosphate solubilization by all tested strains, except for *Phyllobacterium* sp., which recorded the highest phosphate solubilization with ammonium nitrate. The IAA production by the tested strains indicated that *Rhizobium* sp. produced the highest amount of IAA (90.21 μg ml^-1^) in culture media supplemented with L-tryptophan. The best production was observed with L-Trp concentration of 0.2% (116.42 μg ml^-1^) and at an initial pH of 9 (116.07 μg ml^-1^). The effect of NaCl on IAA production was tested at concentrations of 0 to 5% and the maximum production of  89.43 μg ml^-1^ was found at 2% NaCl. The extraction of crude IAA from this strain was done and purity was confirmed with Thin Layer Chromatography (TLC) analysis. A specific spot from the extracted IAA production was found to correspond with a standard spot of IAA with the same Rf value. Finally, the tested PGPR demonstrated growth stimulatory effects on *Acacia cyanophylla* seedlings in vivo, with a great increase of shoots’ and roots’ dry weights, and shoot length compared to control.

The rhizobacterial isolates were identified by 16S rDNA sequence analysis as *Agrobacterium* sp. NA11001, *Phyllobacterium* sp. C65, *Bacillus* sp. CS14, and *Rhizobium* sp. V3E1.

**Conclusion:**

This study highlights the importance of the use of phosphate solubilizing and IAA producer microorganisms as biofertilizers to increase crop yields. The studied strains showed a significant phosphate solubilization potential and IAA production. The use of selected strains as inoculants would be interesting, in particular with a view of promoting sustainable agriculture. However, further studies to verify the efficacy of the best isolates in situ is certainly required.

## Background

Plant growth-promoting rhizobacteria (PGPR) are naturally occurring soil bacteria that can improve plant growth by different mechanisms such as phosphate solubilization, synthesis of plant growth hormones, biological nitrogen fixation, 1-Aminocyclopropane-1-carboxylate deaminase and siderophore productions, and antifungal activity [1–3]. In addition, some PGPR strains can indirectly protect plants by stimulating inducible defense mechanisms (induced systemic resistance), which can make the host much more resistant to future pathogenic aggression [4].

The majority of plant species are associated with PGPR which mainly belong to the following genera: *Acinetobacter, Agrobacterium, Aeromonas, Alcaligenes, Arthrobacter, Azoarcus, Azospirillum, Azotobacter, Bacillus, Beijerinckia, Bradyrhizobium, Caulobacter, Chromobacterium, Derxia, Enterobacter, Erwinia, Flavobacterium, Frankia, Herbaspirillum, Hyphomicrobium, Klebsiella, Micrococcus, Pseudomonas, Rhizobium, Serratia, Stenotrophomonas, Thiobacillus, Xanthomonas, and Zoogloea* [5]. These bacteria may be found in the soil either in symbiotic association with the host plant or in free living forms. Moreover, their potential in agriculture has steadily increased since it represents an alternative strategy to replace the use of chemical fertilizers, pesticides, and other supplements [6]. Hence, introduction of PGPR including nitrogen fixing and phosphate solubilizing bacteria (PSB) as biofertilizers is proposed as a sustainable option in order to improve nutrient availability, plant growth, and yield [7]**.** Indeed, rhizobia were commercialized as inoculants worldwide, mainly in developed countries, in order to enhance plant growth [8]. Acacia is a legume tree which plays an important role in increasing the productivity potential of uncultivable soils due to its capacity to colonize degraded areas and protect the soil against erosion, dune formation, and desertification [9]. The ability of acacia to develop a double symbiotic association with symbiotic nitrogen-fixing bacteria (rhizobia) on the one hand and with endomycorrhizogenic fungi on the other hand gives them socio-economic and ecological importance. Acacia plantations cover an area of 1,128,000 ha of natural forests in Morocco and are among the fast-growing, N_2_-fixing multipurpose woody legumes which are prominent among the exotic species planted in arid and semiarid lands [10]. This tree covers different areas such as the Atlantic Plain (Mediterranean climate), the Middle Atlas (relatively humid), and Souss, presahara, and Sahara arid regions [10]. Moreover, *Acacia cyanophylla* Lindl. (*Acacia saligna* (Labill.) H.L. Wendl) is a native species to arid and subhumid zones of west Australia and South Africa. It can reach 5 to 8 m high and it is considered as the most promiscuous host, for the N_2_ fixing bacteria, since it is efficiently nodulated with a great diversity of rhizobia, including fast-growing taxa such as Rhizobium, Ensifer, and Mesorhizobium and slow-growing ones such as Bradyrhizobium [11].

The reason for focusing on phosphorous is that it is considered, behind nitrogen, as the most important macronutrient which plays a major role in the plants’ growth and the repair of cells and tissues. Phosphorus supports root development in various metabolic processes [12]. Though plants are not able to absorb the insoluble form of phosphorus, they gain the ability through the intervention of the phosphatase enzyme which converts it into soluble forms. Several PSBs have the ability to convert insoluble phosphates into soluble forms bioavailable through the acidification process, chelation, and production of organic acids such as acetic gluconic acid, glycolic acid, malonic acid, isobutyric, isovaleric, and lactic acid etc. [13]. These bacteria are also known to produce growth-promoting substances such as the plant hormone IAA, which is considered as one of the most physiologically active phytohormones that improve plant growth [14]. Indeed, IAA synthesized by rhizobacteria mainly affects the root system by increasing its size, weight, and root hairs and lateral root numbers [15, 16] as well as the soil contact area. This helps to improve the plants’ development and yield by increasing the soil resources use and enhancing the nutrients research and acquisition [17]. Several environmental factors can influence the IAA biosynthesis, in particular, a high pH and the presence of large amounts of tryptophan which lead to an increase in its production [18, 19]. L-tryptophan (L-Trp) is considered as the precursor which enhances microbial biosynthesis of IAA in soil from root exudates or decaying cells [20].

In the present study, we aimed to isolate PGPR strains from *Acacia cyanophylla* root nodules for screening and optimization of indole-3-acetic acid production and phosphate solubilization and then to investigate the inoculation effect with the isolated and identified rhizobacteria on plants’ growth and development in vivo.

## Methods

### Isolation of bacteria

The formal identification of the plant material used in this study was carried out by Pr Kawatr Fikri-Benbrahim. Root nodules were freshly harvested from *Acacia cyanophyll*a growing in sandy loam soil from different regions of Morocco (Table 1) and rinsed delicately with running tap water. The nodules were surface sterilized using 90% ethanol for 10 s and 2% sodium hypochlorite for 5 min and rinsed thereafter three times in sterilized distilled water [21]. The sterilized nodules were aseptically crushed with a sterile glass rod in few drops of NaCl (9 g L^-1^). The bacteria were grown and isolated on yeast extract mannitol agar (YMA) [21]. All isolates were incubated for 3 to 7 days at 28 °C and then stored at − 80 °C in 20% glycerol until further use [22, 23].

**Table 1:** Geographic origin, geodesic coordinates, and climate type of the sampling sites

Isolates	Geographic origin	Latitude N Longitude W Altitude m	Climate type^a^	Average temperature (°C)^a^	Average rainfall (mm)^a^
***Agrobacterium*** **sp.**	Fez	34° 03′ 00′′ N 4° 58′ 59′′ W et 579 m	CSa (Mediterranean climate)	18	536
***Phyllobacterium*** **sp.**	Oujda	34° 41′ 12′′ N 1° 54′ 41′′ W 450 m	BSk (Cold semi-arid climate)	16.7	338
***Bacillus*** **sp.**	Tanger	35° 46′ 02 ′′ N 5° 47′ 59′′ W 20 m	CSa (Mediterranean climate)	17.9	762
***Rhizobium*** **sp.**	Casablanca	33° 35′ 17′′ N 7° 36′ 40′′ W 27 m	CSa (Mediterranean climate)	17.7	412

^a^Climate type of each region according to Köppen climate classification system

### Phosphate solubilization ability of bacterial isolates on agar plates

The selected bacterial isolates were screened for their phosphate solubilizing ability on Pikovskaya (PVK) and National Botanical Research Institute’s phosphate (NBRIP) growth media containing 0.5% of tricalcium phosphate (Ca_3_(PO_4_)_2_) as the source of insoluble phosphate [24]. After incubation at 28 ± 2 °C for 7 days, the diameter of the clear zone which formed surrounding the colony was measured and the solubilizing efficiency (SE%) was expressed according to Srivastav et al. [25] as follows:

$$ \mathrm{Solubilization}\ \mathrm{Efficiency}\ \left(\mathrm{SE}\%\right)=\frac{\mathrm{Diameter}\ \mathrm{of}\ \mathrm{solubilization}\ \mathrm{zone}-\mathrm{Diameter}\ \mathrm{of}\ \mathrm{the}\ \mathrm{colony}}{\mathrm{Diameter}\ \mathrm{of}\ \mathrm{the}\ \mathrm{colony}}\times 100 $$

### Estimation and optimization of phosphate solubilization in liquid culture

Erlenmeyer flasks (250 ml) containing 50 ml of NBRIP or PVK broth media [26] at a neutral initial pH were inoculated in triplicate by 100 μl of inoculum (at ~ 3 10^8^ CFU.ml^-1^), then incubated at 28 ± 2 °C on a rotary shaker (150 rpm) for 7 days. The cultures were harvested by centrifugation at 5000 rpm for 20 min. The final pH of the supernatant was measured, and the P_2_O_5_ liberated was determined according to the method of Jackson [27].

To investigate the effect of nitrogen source on the ability of isolates to solubilize phosphates on liquid medium, different sources were tested. For this reason, ammonium sulfate was replaced with one of the following nitrogen sources: ammonium nitrate, sodium nitrate, potassium nitrate, and asparagine (0.1%). Ammonium sulfate of the standard NBRIP’s medium was considered as control.

The effect of different carbon sources on tricalcium phosphate (TCP) solubilization was also evaluated by replacing the glucose with fructose, inositol, lactose, and mannitol, at a concentration of 1%. These sugars were sterilized separately and were added aseptically to the medium before inoculation. The standard NBRIP’s medium containing glucose was used as a control.

### Screening of bacterial isolates for IAA production

The bacterial isolates were screened for IAA production by using the Salkowski colorimetric assay according to the protocol proposed by Bric et al. [28]**.** The bacterial isolates were cultured in Erlenmeyer flasks (250 ml) containing 50 ml of YMB supplemented with 1 g L^-1^ L-tryptophan at 28 ± 2 °C for 7 days with a shaking speed of 150 rpm in an orbital shaking incubator. Afterwards, the bacterial cultures were centrifuged at 10.000 rpm for 10 min at 4 °C and 1 mL of each supernatant was mixed with 2 mL of Salkowski reagent (1 ml of 0.5 mol L^-1^ FeCl_3_ and 49 ml of 35% HClO_4_). The mix was incubated for 30 min in the dark at 28 ± 2 °C, and then the absorbance was measured at 530 nm. The concentration of IAA produced was estimated using a standard IAA curve. All IAA determinations were made in triplicate.

### Characterization of bacterial isolates by 16s rDNA sequencing

Total genomic DNA from bacterial isolates was extracted by a quick cell lysis in boiling water bath [29]. The nucleotide sequences of the 16S rRNA gene (rDNA) were determined by direct sequencing of appropriate PCR products. A DNA region corresponding to nucleotides 8 to 1540 of 16S rDNA gene rrnB was amplified from each strain with the universal primers 8F (5′-AGA GTT TGA TCC TGG CTC AG-3′) and 1540 R (5′-AAG GAG GTG ATC CAG CC-3′) [30]. Each 50 μl reaction contained 1 μl of the cell lysate (approx. 20 ng DNA), 1.25 U of GoTaqR G2 DNA polymerase (Promega), 1X reaction buffer, 0.2 mM dNTPs, 0.15 μM of each primer, and 5% DMSO. The PCR protocol was set to 4 min at 95 °C for initial denaturation, 35 cycles of amplification: 1 min at 95 °C for denaturation, 1 min at 55 °C for annealing, 2 min at 72 °C for elongation; and the final elongation step was set to 7 min. The nucleotide sequence of the PCR products was determined for both strands by Sanger sequencing. DNA sequences were compared to the GenBank database by basic local alignment search tool (BLAST) requests using the blast-n algorithm and optimization for highly similar sequences (megablast).

### Optimization of IAA production by *Rhizobium* sp.

#### Effect of L-tryptophan concentration

The effect of L-Trp concentration on IAA production by *Rhizobium* sp. was studied. This strain was grown in test Erlenmeyer flasks (250 ml) containing 50 ml of YMB with different concentrations of L-tryptophan (0.1, 0.2, 0.5, 0.7, and 0.9% (v/v)) and incubated at 28 ± 2 °C on a rotary shaker (150 rpm) for 7 days [13]. After incubation, cultures were centrifuged and IAA production was measured and estimated for each culture at 530 nm using Salkowski reagent.

#### Effect of pH on IAA production

To evaluate the effect of pH on the production of IAA, *Rhizobium* sp. was grown in nutrient broth supplemented with 1 g L^-1^ of tryptophan, adjusted to different pH values (5, 6, 7, 8, and 9), and incubated at 28 ± 2 °C on a rotary shaker at 150 rpm for 7 days [14]. The production of IAA was checked after culture centrifugation using Salkowski reagent.

#### Effect of salinity on IAA production

*Rhizobium* sp. was cultured in YMB supplemented with 1 g L^-1^ L-tryptophan with different NaCl concentrations (0, 1, 2, 3, 5% (v/v)) and incubated at 28 ± 2 °C on rotary shaker (150 rpm) for 7 days [31, 32]. IAA produced was measured and estimated after centrifugation for each culture at 530 nm using Salkowski reagent.

#### Effect of incubation period on IAA production

The effect of incubation time was studied in *Rhizobium* sp. in YMB supplemented with 1 g L^-1^ L-tryptophan. Samples were withdrawn every 24 h during the 15 days of incubation at 28 ± 2 °C on a rotary shaker at 150 rpm [33], and the culture supernatant was collected to estimate the IAA produced at 530 nm using Salkowski reagent.

#### Extraction of crude of IAA

Bacterial colony of the best IAA producing rhizobacteria was inoculated into 200 ml of nutrient broth amended with 1 g L^-1^ L-tryptophan and incubated at 28 ± 2 °C on an orbital shaking incubator for 10 days. After incubation, the supernatant was separated by centrifugation at 10,000 rpm for 30 min, acidified to pH 2 with 1 N HCl, and then extracted twice with ethyl acetate. This extracted fraction was evaporated in a rotator evaporator at 40 °C and then the extract was dissolved in 1 ml of methanol and kept at 20 °C.

#### Detection of IAA by thin-layer chromatography (TLC)

Extracts of crude IAA were spread on TLC plates (Silica gel Gf 254, thickness 0.25 mm) and developed using an organic solvent system composed out of n-butanol:ethyl acetate:ethanol:water (3:5:1:1). Spots with Rf values which coincide with that of authentic IAA were identified under UV light (254 nm) by spraying Ehmann reagent on the TLC plates [34].

#### Effect of rhizobacterial inoculation on plant growth

The effect of the studied bacterial isolates on *Acacia cyanophylla* growth was determined in pot experiment using sterilized sandy – loam soil under greenhouse conditions. For this, the seeds were obtained from the High Commission for Water and Forests and the Fight against Desertification of Fez - Boulemane region (http://www.eauxetforets.gov.ma). Surface sterilized and germinated seeds were inoculated with 1 ml of the bacterial suspension of each isolate, grown on YEMB, in the exponential growth phase. The plants were harvested after 6 months, and morphological characteristics including shoot and root dry weights and aerial part length were determined. Non-inoculated plants were considered as control.

### Statistical analysis

The results were statistically analyzed by one-way ANOVA test, carried out using statgraphics centurion XVI. Data were considered as statistically different at the 0.05 significance level.

## Results

### Solubilization of tricalcium phosphate (TCP) by rhizobacterial strains

The phosphate solubilization test showed the ability of the four tested rhizobacterial strains to produce clear zones of phosphate solubilization on PVK and NBRIP agar medium after 7 days of incubation (Table 2). Solubilization efficiency (SE) for each strain was essentially equivalent in the two culture solid media and ranged between 66 and 200%. Maximum efficiency and maximal P. solubilization were observed for *Bacillus* sp. with P_2_O_5_ liberation of 519 μg ml^-1^ in PVK and 782 μg ml^-1^ in NBRIP liquid media. The one-way ANOVA test has shown a significant difference in solubilization efficiency and P_2_O_5_ liberated for the four tested rhizobacterial strains in the two studied media (*p* value < 0.05).

**Table 2:** Solubilization of tricalcium phosphate by the tested rhizobacterial strains

Isolates	PVK medium			NBRIP medium		
	Solubilization efficiency (SE%)	Final pH	P_2_O_5_ liberated (μg ml^-1^)	Solubilization efficiency (SE%)	Final pH	P_2_O_5_ liberated (μg ml^-1^)
***Agrobacterium*** **sp.**	66 ± 1.41	5.95 ± 0.04	298 ± 9.89	66 ± 2.82	5.69 ± 0.02	488 ± 11.13
***Phyllobacterium*** **sp.**	96 ± 2.82	5.8 ± 0.03	410 ± 8.48	91 ± 2.12	5.2 ± 0.04	576 ± 8.48
***Bacillus*** **sp.**	200 ± 4.94	5.28 ± 0.07	519 ± 9.19	190 ± 3.53	4.65 ± 0.07	782 ± 9.19
***Rhizobium*** **sp.**	183 ± 4.94	5.4 ± 0.02	480 ± 12.72	183 ± 2.12	4.85 ± 0.04	684 ± 5.65

Correlation coefficient between final pH and P_2_O_5_ liberated (*r* = − 0.95 in PVK; *r* = − 0.98 in NBRIP); between solubilization efficiency and P2O5 liberated (*r* = 0.95) in both media

Hence, higher P_2_O_5_ amounts were released in NBRIP broth than in PVK broth for all strains. Moreover, a pH decrease was noted concomitantly with phosphate solubilization. The statistical analysis revealed a positive correlation between the solubilization efficiency on agar media and P_2_O_5_ liberated in broth (*r* = 0.95). Also, a negative correlation was observed between the P_2_O_5_ liberated and the final broth’s pH (*r*_1_ = − 0.95 in PVK; *r*_2_ = − 0.98 in NBRIP).

### Effect of carbon and nitrogen sources on phosphorus solubilization

The effect of carbon source on phosphorus solubilization was evaluated on tested strains using five different carbon sources. The obtained results showed that glucose induced the best TCP solubilization, when used as a carbon source, while inositol induced the lowest TCP solubilization (Table 3). In this study, pH decrease was clearly observed alongside an increase in phosphate solubilization, independently of the carbon sources tested. Statistical analysis showed that the effect of different carbon sources on TCP solubilization by the tested strains was significant (*p* value < 0.05).

**Table 3:** Effect of different carbon sources on the tricalcium phosphate solubilization by the tested rhizobacterial strains

Carbon source (1%)	***Agrobacterium*** sp.		***Phyllobacterium*** sp.			***Bacillus*** sp.	***Rhizobium*** sp.	
	Final pH	P_2_O_5_ liberated (μg ml^-1^)	Final pH	P_2_O_5_ liberated (μg ml^-1^)	Final pH	P_2_O_5_ liberatd (μg ml^-1^)	Final pH	P_2_O_5_ liberated (μg ml^-1^)
**Glucose (control)**	5.69 ± 0.16	488 ± 8.48	5.22 ± 0.02	576 ± 5.65	4.65 ± 0.04	782 ± 9.19	4.85 ± 0.04	684 ± 6.36
**Fructose**	6.23 ± 0.03	240 ± 5.65	6.22 ± 0.05	256 ± 6.36	5.78 ± 0.04	320 ± 10.6	6.65 ± 0.08	365 ± 12.02
**Mannitol**	6.60 ± 0.08	140 ± 10.6	6.76 ± 0.04	123 ± 9.19	6.18 ± 0.04	250 ± 7.07	6.22 ± 0.02	232 ± 5.65
**Lactose**	6.88 ± 0.22	92 ± 4.24	6.72 ± 0.66	124 ± 6.36	6.48 ± 0.09	174 ± 8.48	6.28 ± 0.04	256 ± 4.24
**Inositol**	6.55 ± 0.14	160 ± 4.24	6.86 ± 0.04	94 ± 5.65	6.91 ± 0.08	74 ± 9.89	6.88 ± 0.05	120 ± 10.60

The effects of different nitrogen sources on phosphate solubilization by the tested strains were reported in Table 4. High phosphate solubilization was detected by *Bacillus* sp. (782 μg ml^-1^) when ammonium sulfate was used as a nitrogen source, with the highest pH decrease. However, for *Phyllobacterium* sp., the highest phosphate solubilization was recorded with ammonium nitrate as a nitrogen source (580 μg ml^-1^). The pH reduction was noticed for all strains with the different used nitrogen sources. In addition, the best TCP solubilization was observed with inorganic nitrogen rather than with the organic N sources, for all tested strains.

**Table 4:** Effect of different nitrogen sources on the tricalcium phosphate solubilization by the tested rhizobacterial strains

Nitrogen source (0.1%)	***Agrobacterium*** sp.		***Phyllobacterium*** sp.		***Bacillus*** sp.		***Rhizobium*** sp.	
	Final pH	P_2_O_5_ liberated (μg ml^-1^)	Final pH	P_2_O_5_ liberated (μg ml^-1^)	Final pH	P_2_O_5_ liberated (μg ml^-1^)	Final pH	P_2_O_5_ liberated (μg ml^-1^)
**Ammonium sulfate (control)**	5.69 ± 0.02	488 ± 5.65	5.22 ± 0.05	576 ± 6.36	4.65 ± 0.04	782 ± 4.24	4.85 ± 0.04	684 ± 5.65
**Sodium nitrate**	5.82 ± 0.04	310 ± 2.82	6.21 ± 0.02	250 ± 6.36	5.72 ± 0.02	455 ± 9.19	5.75 ± 0.02	420 ± 9.19
**Potassium nitrate**	5.85 ± 0.04	302 ± 7.07	6.18 ± 0.04	253 ± 3.53	5.82 ± 0.04	320 ± 12.02	6.22 ± 0.04	235 ± 4.94
**Ammonium nitrate**	5.74 ± 0.05	420 ± 11.31	4.95 ± 0.04	580 ± 10.6	4.72 ± 0.02	755 ± 5.65	4.92 ± 0.04	588 ± 11.31
**Asparagine**	6.52 ± 0.04	185 ± 4.94	6.78 ± 0.05	92 ± 8.48	6.90 ± 0.07	68 ± 2.12	6.89 ± 0.21	96 ± 6.36
**Urea**	6.75 ± 0.05	95 ± 7.07	6.88 ± 0.08	74 ± 7.07	6.95 ± 0.05	44 ± 4.94	6.86 ± 0.02	78 ± 4.94

### Determination of IAA production by rhizobacterial strains

The results of IAA production were found positive for all the tested rhizobacteria. In fact, the highest concentration of IAA (90.21 μg ml^-1^) was produced by *Rhizobium* sp. while the lowest production was seen by *Phyllobacterium* sp. (67.85 μg ml^-1^), whereas *Bacillus* sp. and *Agrobacterium* sp. were able to produce 83.54 μg ml^-1^ and 74.3 μg ml^-1^ of IAA, respectively.

### Optimization of IAA production

**Fig. 1:**
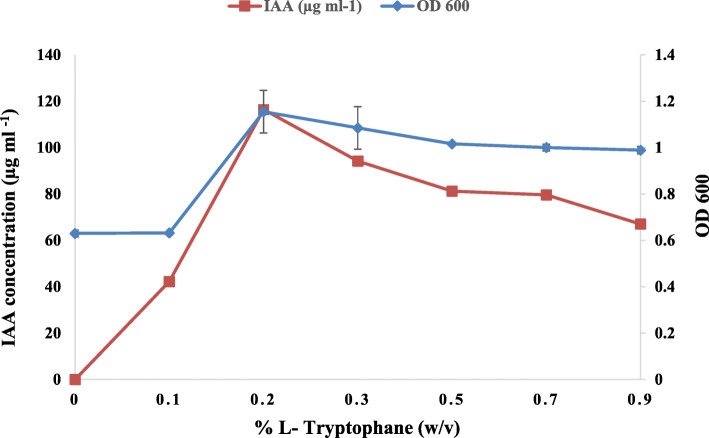
Effect of L-tryptophane concentrations on *Rhizobium sp.* growth and IAA production

**Fig. 2:**
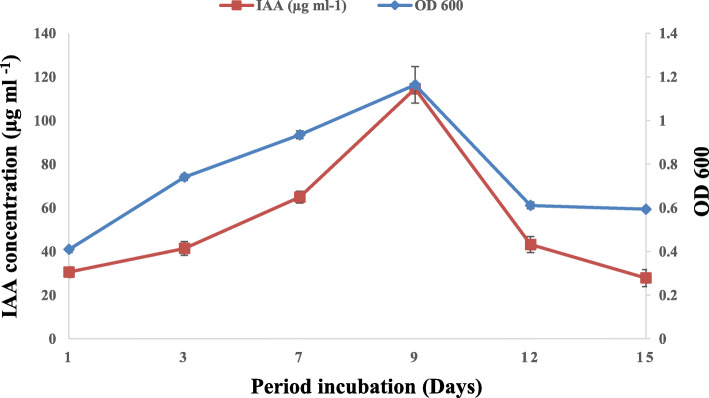
Effect of incubation period on *Rhizobium sp*. growth and IAA production

**Fig. 3:**
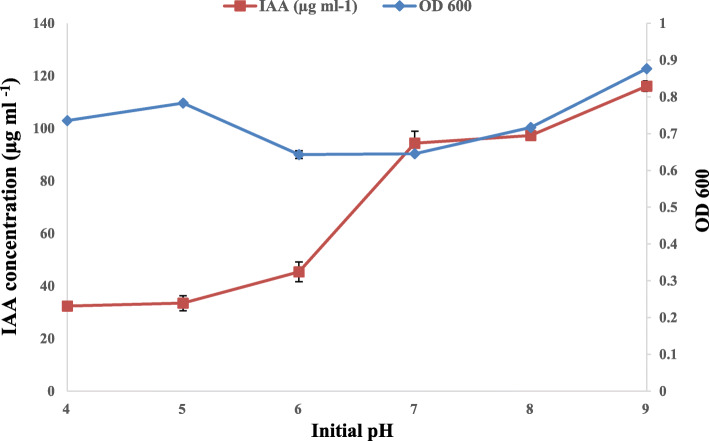
Effect of different pH values on *Rhizobium sp*. growth and IAA production

**Fig. 4:**
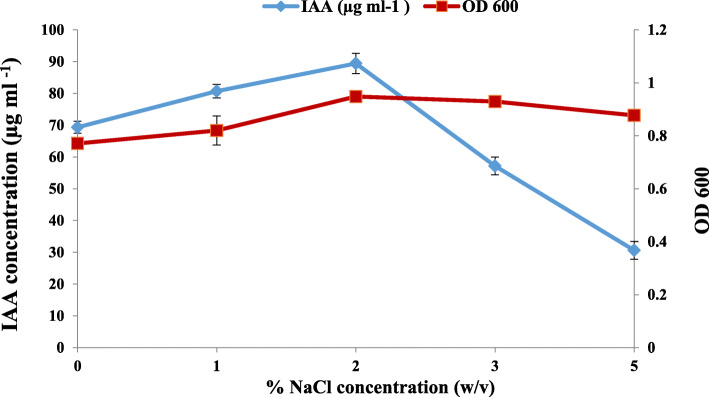
Effect of NaCl concentrations on *Rhizobium sp.* growth and IAA production

Tryptophan concentration affected the IAA production; in fact, the increase in L-Trp concentration induces an increase in the amount of IAA produced. The best production was observed with concentrations of 0.2% L-Trp. At a higher concentration of L-Trp, a decrease in the amount of produced IAA was detected (Fig. 1). The IAA production by *Rhizobium* sp. started with bacterial growth and increased steadily before reaching a maximum after 9 days of incubation. However, the level of IAA production decreased after 12 days of incubation (Fig. 2). This can be explained by the degradation of IAA by the bacteria. The effect of pH on IAA production was determined at different pH values ranging from 4 to 9 (Fig. 3). The maximum IAA production by the studied strain has been detected at pH 9 (116.07 μg ml^-1^), but this production decreased with the pH decrease. Moreover, the maximum production of IAA (89.43 μg ml^-1^) was obtained at 2% NaCl (Fig. 4), but higher NaCl concentrations seem to have an inhibitory effect on the bacterial growth and IAA production.

The production of IAA from Trp by *Rhizobium* sp. was confirmed and compared with standard IAA on TLC chromatograms. TLC of ethyl acetate extracts showed a clear pink-colored spot at the Rf corresponding to the authentic IAA (0.5), when chromatograms were treated with Ehmann’s reagent.

### Effect of rhizobacteria on plants’ growth

**Fig. 5:**
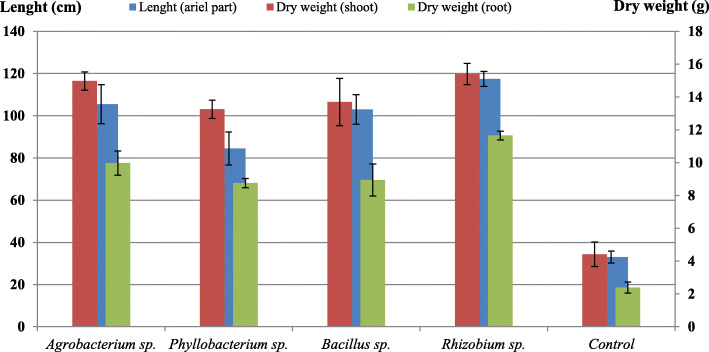
Effect of inoculation with rhizobacterial strains on *Acacia* sp. growth

The strains were evaluated subsequently for their ability to form a beneficial association with *A. cyanophylla*. Results obtained during this study showed a positive effect of inoculation by the tested rhizobacteria on the plant growth (Fig. 5). In fact, *Rhizobium* sp. exhibited the highest increase in shoot and root dry weights and in shoot length by 3.49, 4.89, and 3.56 times, respectively, compared to control. These results were confirmed by statistical analysis using a one-way ANOVA test which revealed a statistically significant difference between studied rhizobacteria and control (*p* value < 0.05).

### Molecular classification of the plant growth-promoting isolates

The sequencing of 16S rRNA genes of bacterial isolates was repeatedly reported as the preferred method for characterizing microbial communities, found in the plant rhizosphere [35]. In this study, the four analyzed isolates could be clearly assigned to their “species group,” namely *Rhizobium* sp., *Phyllobacterium* sp., *Bacillus* sp., and *Agrobacterium* sp. (Table 5). The BLAST homology searches showed precise matches with sequences of the corresponding strains in GenBank (99%), both for sequences resulting from forward and from reverse sequencing reactions.

**Table 5:** Sequence analysis of 16S rDNA for rhizobacterial strains associated with root nodule of *Acacia cyanophylla*

Isolate	Accession (no)	Homology to the Identity reference strains	Identity (%)
**1**	KJ648176.1	*Phyllobacterium* sp. C65	99
**2**	MT584789.1	*Bacillus* sp. CS14	99
**3**	AB921256.1	*Agrobacterium* sp. NA11001	99
**4**	MG637030.1	*Rhizobium* sp. V3E1	99

## Discussion

Isolation and selection of efficient rhizospheric strains nodulating legumes are important for the development of biotechnology products that can be used in inoculation field trials. The result in Table 2 highlights the existence of phosphate solubilizing bacteria nodulating acacias trees. The studied isolates were able to solubilize insoluble phosphate in both PVK and NBRIP media. However, this latter showed higher efficiency compared to the first culture media. Comparative tests on PVK and NBRIP agars showed almost similar results concerning the phosphate solubilizing capacity. This result is concomitant with that found by Nautiyal [26]. In general, it was observed that phosphate solubilization was accompanied by a decrease in the medium’s pH. Among all tested carbon sources on the phosphate solubilization ability of rhizobacterial isolates, glucose induced the greatest increase in total soluble phosphate. Hence, the maximum phosphate solubilization was generally observed when the glucose was used. These results were consistent with previous studies which reported that glucose was the best carbon source for phosphate solubilization [36–38].

The effect of different nitrogen sources on phosphate solubilization activity of the studied strains was indicated in Table 4. The results showed that the highest phosphate solubilization was recorded in the presence of ammonium sulfate for most strains followed by ammonium nitrate. This solubilization was always accompanied by a decrease in pH. This negative correlation between phosphate solubilization by tested isolates and the pH of culture medium was previously reported by other studies [37, 39]. During solubilization, microorganisms can transform insoluble phosphates into soluble forms through a chelation process, or acidification of periplasmic space by direct oxidation of glucose. This leads to the release of different organic acids, such as acetic, gluconic, glycolic, isobutyric, isovaleric, lactic, malonic, oxalic, and succinic acids. This process mobilizes phosphoric components, especially with inorganic fertilizers added to the soil [13]. However, these compounds are hardly released in solid medium unlike liquid medium [26].

Phosphate solubilizing microorganisms (PSM) are considered as a better means of ecological nutrition for plant crops. Inoculation of soil or seeds by these microorganisms is known to improve solubilization of soil-fixed phosphorus and of applied phosphate, which can therefore increase plant yield [40]. It has also been reported that PSM can help release additional phosphate into soil solution once their metabolic needs have been satisfied [13].

IAA is considered to be one of the most important phytohormones whose production by PGPR can vary among different species and strains, and which is influenced by culture conditions, growth stage, and substrate availability [41]. In this study, all the rhizobacterial strains showed a positive result for IAA production. Under natural conditions, plant roots excrete organic compounds, including L-Trp, which can then be used by the rhizobacteria for IAA biosynthesis. Several pathways have been reported for the conversion of Trp to IAA by rhizobacteria. In fact, it was reported that the conversion of Trp to IAA can involve deamination, decarboxylation, and/or hydrolysis reactions. The indole-3-pyruvic acid (IpyA) pathway is the primary pathway for IAA synthesis, whereas other pathways operate in some species (the indole-3-acetamide pathway, the tryptamine pathway, and the indole-3-acetonitrile pathway) [42]. The IAA production by rhizobacteria can help non-native plant species to resist biotic and abiotic stress conditions. However, little information is available on the relationship between stress and auxins in plants, and the evolutionary role played by auxin in the adaptation of plants to various environmental stresses [18].

The effect of different L-Trp concentrations revealed that maximum growth and IAA production (116.42 μg IAA ml^-1^) were observed with L-Trp concentrations of 0.2% (Fig. 1). Similarly, a *Rhizobium* sp. isolated from root nodules of a leguminous shrub, *Cajanus cajan*, has been reported to produce a maximum amount of IAA at 0.2% of L-Trp [43]. It is observed that IAA production by *Rhizobium* sp. has been steadily increasing over time before reaching a maximum of 114.6 μg IAA ml^-1 ^after 9 days of incubation and then decreased slowly. This result is consistent with an earlier study that showed that the maximum production of IAA was achieved after 9 days of incubation [13]. The decrease in IAA production might be due to the release of IAA degrading enzymes such as IAA oxidase and peroxidase, as has been reported earlier in some species of *Rhizobium* bacteria [13, 44].

The effect of pH variation on IAA production showed that *Rhizobium* sp. produced more IAA at pH 9, but this production decreased with the pH decrease. Similarly, previous studies showed that the maximum yield of IAA was obtained at pH 9 [14, 45]. Other authors found that the maximum amount of IAA was produced at neutral to slightly alkaline pH [37, 46, 47].

The effect of various concentrations of NaCl indicated that the higher amount of IAA produced was obtained at 2% NaCl (Fig. 4), but higher NaCl concentrations seem to have an inhibitory effect on the bacterial growth and IAA production. This is concordant with Wang et al. findings [48], which reported that the production of IAA declined in response to salinity. However, there are few reports on IAA production under salt stress.

PGPR have been known for their beneficial effects on plants such as the obvious increase in the weight and length of their aerial and root parts. In this study, the tested rhizobacteria revealed desirable effects on *A. cyanophylla* growth, which caused significant variations between the studied parameters compared to control. Rhizobia strains can promote seed emergence and seedling germination which is beneficial for early seedling establishment and consequently the crop growth and development [49]. In fact, IAA acts as a signal molecule involved in plant signal processing, motility, or fixing bacteria in the roots which helps to promote legume-rhizobium symbiosis [50]. It is reported that *Bacillus* species can affect positively plant growth by enhancing significantly the shoot length and total dry weight. This effect was related to the production of phytohormone and siderophore and the ability of these bacteria to solubilize phosphate [51, 52]. A previous study confirmed that *Agrobacterium rhizogenes* (TR105) was also very effective in increasing jujube’s root length (*Watkins jujube*) and has great rooting potential [53]. Thus, Ipek et al*.* [54] showed that *Agrobacterium A18* may be potentially used as PGPR.

*Phyllobacterium* is a genus originally isolated from leaf nodules of some plant families. It contains several recently described species associated with plant roots and endophytes of legume nodules [55, 56]. *Phyllobacterium brassicacearum* strain STM196 isolated from canola rhizospheres has shown its ability to promote canola and Arabidopsis growth in soil and in vitro. In vitro STM196 inoculation stimulated root hair elongation and increased the total length of lateral roots [57].

The presence of a P-solubilizing and IAA-producing microbial population in soils may be considered as a positive indicator for the use of microorganisms as biofertilizers for crop production in sustainable agriculture. In fact, the use of such kinds of bacteria is proposed as a key to promoting plant growth and increasing yield [58]. Indeed, the inoculation of PGPR with multifunctional features is better than the inoculation of those having unique traits [59].

The phosphate solubilizing strains can increase soil phosphorus availability to the plant by mineralizing organic phosphorus compounds and converting inorganic phosphorus to a more available form. IAA plays a key role in the root cell division’s initiation, cell enlargement, and increase in root surface area, and therefore improves access to soil nutrients through better root formation [35, 60].

Acacias are characterized by good growth in drylands having low water and nutrient availability and play a very important role in maintaining the ecological and hydrological stability of native arid and semi-arid ecosystems [29]. Some acacia species are widespread in Africa and are efficiently nodulated with fast and slow-growing rhizobia [61]. The ecological interaction between the acacias and rhizobacteria has a beneficial potential, which results in the increase of biomass and the improvement of degraded sites through a better absorption of water and nutrients, a prevention of soil erosion and a soil fertility increase by fixing N_2_ and producing nutrients [62]. The identified species can be found ubiquitously in the plant rhizosphere and have all been reported to promote growth to, e.g., barley, wheat, maize, potatoes, legumes, vegetables, and forage crops [2, 63–68]. So, our tested strains have a great potential to be used as inoculants, especially because of their double ability of nodulating Acacia and solubilizing phosphorus. However, further studies to verify the survival and competitiveness of these strains in situ are certainly required to provide sufficient information allowing the use of these PGPR as inoculants.

## Conclusion

This study indicates that there are several beneficial traits in rhizobacterial strains that could improve plant growth. In fact, high phosphate solubilization efficiency was recorded for *Bacillus* sp. with approximately 782 μg ml^-1^ P_2_O_5_ liberated. Maximum solubilization was recorded when glucose was used as carbon source and ammonium sulfate as a nitrogen source for all strains except *Phyllobacterium* sp. which recorded the highest phosphate solubilization with ammonium nitrate. Interestingly, the highest IAA production was detected for *Rhizobium* sp. with an amount of 116.42 μg ml^-1^ at L-Trp concentration of 0.2%. The microbial phosphate solubilization and IAA production can play an important role in making soluble phosphate bioavailable for plants and improving plant growth and development. Indeed, the tested rhizobacteria have validated their potential role to be used as inoculum for improving the growth of *Acacia cyanophylla*. Hence, inoculation by such nitrogen-fixing bacteria having also a phosphorus solubilizing potential could be an excellent alternative to promote plant growth in the absence of chemical fertilization and enable them to better adapt to difficult conditions, especially for acacias, which are nitrogen-fixing plants, often intended for soil restoration and rehabilitation purposes.

## Data Availability

All data generated or analyzed during this study are included in this published article

## Abbreviations

FeCl_3_: Iron trichloride
HClO_4_: Perchloric acid
IAA: Indole-3-acetic acid
NaCl: Sodium chloride
NBRIP: National botanical research institute phosphate
PCR: Polymerase chain reaction
PGPR: Plant growth-promoting rhizobacteria
pH: Potential hydrogen
PSB: Phosphate solubilizing bacteria
PSM: Phosphate solubilizing microorganisms
PVK: Pikovskaya
RPM: Rotation per minute
SE: Solubilization efficiency
TCP: Tricalcium phosphate
TLC: Thin layer chromatography
Trp: Tryptophan
YMB: Yeast-mannitol broth
